# The Conventions

**Published:** 1891-06

**Authors:** 


### Editorial.

### THE CONVENTIONS.

The season for the gathering of the various conventions both in this country and Europe is approaching, and it is important that those interested should arrange to make these worthy the profession they represent. To do this means a wider and deeper interest than has heretofore been manifested both by those who have been active in their development as well as others who usually let them pass by without a thought.

The professional spirit in dentistry needs cultivating, indeed, it may be questioned whether beyond a small circle it has any existence. From that limited number has come the earnest workers who yearly sacrifice time, money, and strength that dentistry may have a living organization to represent its thought and activity.

The lack of interest in the work of the profession in its literature, its meetings, and the innumerable details necessary to progress, has been a serious hinderance to its advancement. The meetings come and go, and the individual members are found either at home, over the chair, or seeking enjoyment and recreation elsewhere, indifferent to the sacrifices being made for their good and that of the entire body.

This indifference is not wholly based on selfish considerations. With many it has had its origin in the organizations and their management. The time was when these national gatherings were looked forward to with an ever-increasing interest. They were regarded as the exponents of the best thought and represented the highest ideals in theory and practice; but in time they have almost ceased to have a controlling influence, and it has come to be regarded by many as a matter of no importance whether the American Dental Association meets or not, as it has ceased to have any power for good or ill to this class or, in their opinion, to the profession at large. If this idea be the true one, and it is feared it cannot be controverted, it is time that the active workers in these various bodies were asking themselves the question, What is the cause and the remedy? This is more easily asked than answered.

The primary consideration is to bring about a union of effort. At the present time there are three national organizations,—the

American Dental Association, the Southern Dental Association, and the Section of Oral and Dental Surgery of the American Medical Association. At the present writing the latter is in session at Washington, D. C. As membership here is confined to those having the degree of Doctor of Medicine, it naturally follows that this must be limited. The American Dental Association is arranged in two classes,—permanent members and delegates. The Southern Dental Association is, we believe, modelled after the same plan.

It is, perhaps, idle to expect the section of the American Medical Association to abandon their exclusive organization, nor is it probable that either of the other two will change their mode of procedure. Yet it is apparent that unless a better system be adopted, the present mode of meeting together will fail eventually from its own dead weight.

It is not the time or place to enter into the consideration of proposed changes. These will come; but only by the influence of a more progressive life in the various bodies. This desirable state can, in our judgment, be best attained by each of the local society organizations sending up a vigorous intelligent body of men as delegates, with instructions and determination to make the American Dental Association at least a representative body, and so change the constitution that the "permanent-membership" clause may be cast into deserved oblivion. This should never have been engrafted on a society that aims to exercise a controlling influence over the polity of the general body. It has figuratively been the millstone around the neck of the Association for years.

Delegates have been sent to represent local societies, and have found that instead of a deserved welcome they have metaphorically been shown the door. It is not pleasant to be informed that as delegates no privileges can be extended, no membership in sections can be permitted, no place among the offices is open, nothing is given but the privilege of paying five dollars and the right to speak upon the subjects under consideration.

It is time that a broader view should be taken than has heretofore prevailed. The effort, which occurs almost annually, to crush expression on the floor from those not members, or who may be considered such, should receive the condemnation of all liberal-thinking men. If a rule be necessary to meet disorder, let it be elastic, so that those who meet there for scientific progress may not be subjected to the unpleasant scenes so often witnessed, and brought about by those who make rules of order paramount to the main purpose of the meeting.

A representative organization, the outgrowth of properly-arranged local societies, is what is most needed at the present time. The best organized State, as far as the writer is aware, is New York, and the system of district associations has given a vigor and impetus to professional work there not equalled in any other State of the Union. Something of a similiar character is needed for the United States at large.

Let every man who can secure the proper credentials go to Saratoga. The expense of living there is not greater than elsewhere if the large hotels be avoided, and, with this difficulty overcome, there is no pleasanter place in the United States for a week's sojourn, or one more healthful.

The organizations which follow the American Dental Association as a matter of convenience—the National Association of Dental Faculties and the National Board of Dental Examiners—will be there; at least, the former has so announced, and it is presumed the latter will find it most satisfactory to do so. It is to be hoped that the National Board will approach the work of this year with a closer circumspection of their proceedings than was manifested at Excelsior Springs. They have a delicate work to do, and to accomplish it without undue friction requires tact of the highest order.

Those who contemplate going abroad should remember that the American Dental Society of Europe meets this year at Heidelberg, on the Necker, August 3, 4, and 5. The reunion, in such surroundings, it may safely be promised, will be a delightful one.

